# A fungal symbiont converts provisioned cellulose into edible yield for its leafcutter ant farmers

**DOI:** 10.1098/rsbl.2022.0022

**Published:** 2022-04-20

**Authors:** Benjamin H. Conlon, David O'Tuama, Anders Michelsen, Antonin J. J. Crumière, Jonathan Z. Shik

**Affiliations:** ^1^ Section for Ecology and Evolution, Department of Biology, University of Copenhagen, Universitetsparken 15, 2100 Copenhagen East, Denmark; ^2^ Section for Terrestrial Ecology, Department of Biology, University of Copenhagen, Copenhagen, Denmark; ^3^ Smithsonian Tropical Research Institute, Apartado Postal 0843-03092, Balboa, Ancon, Panama

**Keywords:** *Atta*, *Leucoagaricus*, gongylidia, herbivory, nutrition, stable isotope

## Abstract

While ants are dominant consumers in terrestrial habitats, only the leafcutters practice herbivory. Leafcutters do this by provisioning a fungal cultivar (*Leucoagaricus gongylophorus*) with freshly cut plant fragments and harnessing its metabolic machinery to convert plant mulch into edible fungal tissue (hyphae and swollen hyphal cells called gongylidia). The cultivar is known to degrade cellulose, but whether it assimilates this ubiquitous but recalcitrant molecule into its nutritional reward structures is unknown. We use *in vitro* experiments with isotopically labelled cellulose to show that fungal cultures from an *Atta colombica* leafcutter colony convert cellulose-derived carbon into gongylidia, even when potential bacterial symbionts are excluded. A laboratory feeding experiment showed that cellulose assimilation also occurs *in vivo* in *A. colombica* colonies. Analyses of publicly available transcriptomic data further identified a complete, constitutively expressed, cellulose-degradation pathway in the fungal cultivar. Confirming leafcutters use cellulose as a food source sheds light on the eco-evolutionary success of these important herbivores.

## 1. Introduction

Cellulose is a major constituent of plant cell walls and the most abundant organic compound on earth, with enormous potential as an energy source in terrestrial ecosystems [1,2]. However, cellulose is also a recalcitrant molecule that is metabolically inaccessible to most animals without help from bacterial or fungal symbionts [2,3]. While leafcutter ants are the only ants to forage fresh vegetation, they cannot directly consume this cellulose-rich material. Instead, the ants use it to provision an obligate fungal symbiont, *Leucoagaricus gongylophorus* (Basidiomycota, Agaricaceae). The fungus converts plant material into structural hyphae and swollen hyphal cells called gongylidia (growing in bundles called staphylae) that are the main food source for the ants [4–6]. Leafcutter farming systems can be massive. Colonies in the genus *Atta* (Hymenoptera, Formicidae) can contain millions of ant workers and are dominant herbivores in neotropical ecosystems [7]. Despite their large-scale herbivory, it remains uncertain whether these farming systems can use recalcitrant plant polymers, like cellulose, as a source of nutrition [8,9] (electronic supplementary material, table S1).

Recent studies have shown the presence of cellulose degradation and cellulose-degrading enzymes in leafcutter fungus gardens [10–17]. However, it remains unclear if these enzymes serve primarily to degrade the cell wall and thus provide access to more readily metabolized nutrients inside, or if the fungus is also capable of assimilating cellulose-derived carbon (C) into edible nutritional rewards (i.e. gongylidia). Cellulose digestion may also be context specific, with the cultivar prioritizing more accessible carbon sources whenever possible [13], as is also reported from other fungal lineages [18]. Alternatively, cellulose degradation has been attributed to bacterial symbionts within the fungus garden rather than the fungus itself [11,16,17], but see [19]. We review the literature on cellulose degradation in the leafcutter symbiosis in electronic supplementary material, table S1.

We tested the links between cellulose provisioning, cellulose metabolism and the cultivar's production of nutritional rewards in three steps. First, an *in vitro* experiment with ^13^C-labelled cellulose measured the uptake of cellulose-derived carbon into hyphae and staphylae. The inclusion of dextrose as a more accessible carbon source in the media provided a secondary test of whether the fungus uses cellulose as an energy source even when simpler sugars are available. Second, an *in vivo* laboratory feeding experiment tested if the *in vitro* results could be replicated in a colony of the Panamanian leafcutter ant *Atta colombica*. Foragers collected agar-based substrate containing ^13^C-labelled cellulose which gardeners used to provision their fungus garden, containing a natural assemblage of ants and microbes. Third, we tested if the fungal cultivar can directly metabolize cellulose in two steps. *In silico* analysis of previously published transcriptomic data assessed if the cultivar constitutively expresses a complete metabolic pathway for cellulose degradation, even when this compound is not expected to be present in the provisioned substrate. An *in vitro* experiment with media containing ^13^C-labelled cellulose and antibiotics assessed cellulose metabolism following targeted bacterial exclusion.

## 2. Methods

### (a) *In vitro* assays

Fresh fungal cultures of *L. gongylophorus* were isolated from a Panamanian *A. colombica* colony (Ac-2012-1) onto potato-dextrose agar (PDA) [20]. Isotopically enriched media were prepared by adding 0.1 g l^−1^ of ^13^C-enriched glucose (d-glucose-1-^13^C, Sigma-Aldrich, USA) or 0.1 g l^−1^ of ^13^C-enriched cellulose (U-^13^C Cellulose, U-10508, Iso*Life*, The Netherlands) to PDA. Media were autoclaved and 10 ml aliquots were transferred into 60 mm Petri dishes (*n* = 30 per treatment). The ^13^C-enriched glucose treatment represented a positive control as its metabolism and assimilation have previously been confirmed [21]. PDA without enriched compounds was the negative control. Inoculation of the fungus followed established protocols with incubation at 23.5°C [20]. Polycarbonate track-etched (PCTE) membrane discs (diameter 47 mm, PCTE 0.1 µm; GVS, USA) were placed in Petri dishes to facilitate collection of fungal tissues for subsequent analyses after 79 days.

A second experiment repeated this approach but added antibiotics (ampicillin, chloramphenicol and streptomycin) (for concentrations, see: [22]) to each treatment (*n* = 15 per treatment) and was performed over 52 days. We confirmed that bacteria were excluded from antibiotic-treated plates by collecting fungal mycelia from the antibiotic-treated and control plates and extracting DNA using a Chelex^®^ (Sigma-Aldrich, USA) protocol [23,24]. DNA for positive controls was extracted, using the same method, from pure colonies of bacteria: *Streptomyces* sp. (Gram-positive) and *Stenotrophomonas* sp. (Gram-negative). DNA extracts were diluted to 10% of the original concentration using ddH_2_O before analyses. Bacterial load was quantified using ddPCR with eubacterial primers (63F and 355R) following established protocols (Bio-Rad, USA) [25,26]. Based on values for the negative controls, a detection threshold of 10 000 was used (electronic supplementary material, figure S2 and table S2).

### (b) *In vivo* assay

Baseline samples of hyphae and staphylae (*n* = 4 per tissue type) were collected from the middle layer of the fungus garden of Ac-2012-1, maintained in the laboratory at 23.5°C [27]. The colony was provided with a ^13^C-cellulose-enriched diet (see *in vitro* assays), which was completely consumed by the ants within 24 h. Hyphae and staphylae were sampled from the middle layer of the garden after 2 days (*n* = 4 per tissue type), the time when peak ^13^C enrichment levels were previously detected [21].

### (c) Testing for ^13^C assimilation

We collected 0.05–0.1 mg (dry mass) of hyphae and staphylae from each *in vitro* plate and each *in vivo* fungal sample. In addition, remaining media from the initial *in vitro* experiment were collected (electronic supplementary material, figure S1). Samples were prepared following established protocols [21] and then analysed by isotope ratio mass spectrometry (IRMS) for ^13^C/^12^C concentrations (^13^C enrichment). The system used a Eurovector CN analyser (Pavia, Italy) coupled with an Isoprime mass spectrometer (Cheadle Hulme, UK). We used the results to calculate ^13^C enrichment (^13^Cµg g^−1^) in the excess of natural abundance. Each cellulose molecule ([^13^C_6_H_10_O_5_]_n_) had a sixfold higher ^13^C enrichment than each glucose molecule (^13^C-C_5_H_12_O_6_), so we corrected for this by dividing ^13^Cµg g^−1^ values in the cellulose treatment by 6 before further analyses. We used Z-scores to normalize enrichment values relative to baseline abundances for each tissue type, allowing for direct statistical comparisons between tissues and carbon sources (electronic supplementary material, table S3).

### (d) Data analysis

All data were analysed in R (v. 4.0.2 [28]). The homogeneity of variance was tested using Levene's test (*car* v. 3.1–10 [29]) and normality was tested using a Shapiro–Wilk test. Based on these results, *in vitro* IRMS data were analysed non-parametrically using permutational analysis of variance (*Adonis* with Euclidean distances and 9999 permutations; *vegan* version: 2.5–7 [30]). *In vivo* IRMS and ddPCR data were analysed using linear models, with *emmeans* (v. 1.7.2 [31]) used to test for between-tissue differences on Day 2 of the *in vivo* experiment. We performed three separate analyses (two *in vitro*, one *in vivo* experiment), using Z-scores, calculated relative to the control for that tissue type, as the dependent variable unless otherwise specified. The independent variables were as follows: *EnrichedCarbonSource* (enriched cellulose, enriched glucose, none control), *Tissue* (hyphae, staphylae) and *AntiobioticTreatment* (±antibiotics). For the first *in vitro* experiment, we tested *EnrichedCarbonSource + Tissue*, and for the second *in vitro* experiment we tested AntiobioticTreatment*Enriched CarbonSource + Tissue. ^13^C enrichment in the media after the experimental period was tested using ^13^Cµg g^−1^ *EnrichedCarbonSource* (electronic supplementary material, figure S1). For the *in vivo* experiment, we tested Tissue*EnrichedCarbonSource to compare enrichment in staphylae and hyphae to the baseline natural abundance. To test for bacterial DNA in the second *in vitro* experiment, we tested *log10(16S_copies)* against *AntibioticTreatment + CarbonSource*. When main effects were significant, we used *pairwiseAdonis* (v. 0.0.1 [32]) to perform pairwise post hoc tests with adjusted *p*-values calculated using *false-discovery rate* with a significance threshold of *p*_adj_ = 0.05.

### (e) *In silico* analysis of capacity for cellulose metabolism

Transcriptome assemblies [33] were downloaded from the NCBI TSA database, translated using *transeq* (EMBOSS v. 6.6.0 [34]) and carbohydrate-active enzymes (CAZymes) annotated using peptide pattern recognition (PPR) (HotPep v. 1.0 [35]). While previous studies have identified CAZy families expressed in the fungus garden and *in vitro* cultures [13,14,33], PPR predicted enzyme commission (EC) numbers, enabling us to identify the specific reactions catalysed [35]. Predicted EC numbers were compared to the BRENDA [36] cellulose-degradation pathway. We identified all enzymes in the BRENDA pathway.

## 3. Results

### (a) *In vitro* cellulose assimilation by the cultivar

**Figure 1.**
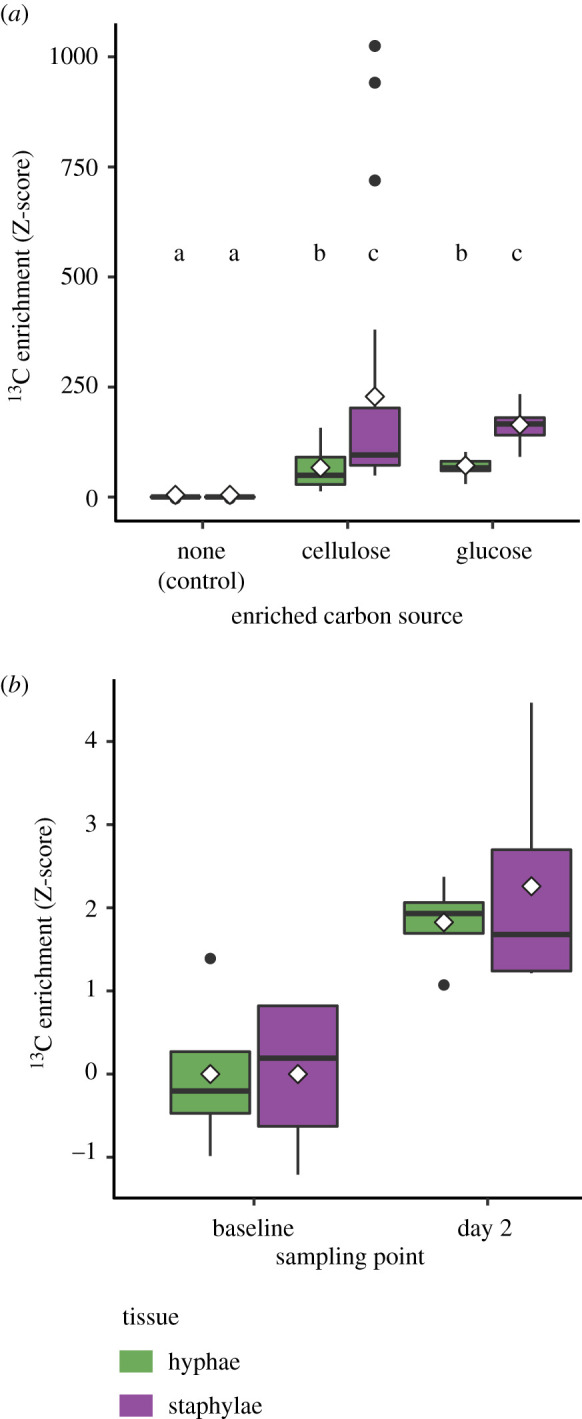
Isotopic evidence that the fungal cultivar assimilates carbon (C) from cellulose molecules. (*a*) An *in vitro* experiment detected significant overall ^13^C enrichment in fungal hyphae and staphylae on ^13^C-enriched media compared to the control. Levels of ^13^C enrichment for staphylae and hyphae did not differ between isotopic enrichment treatments but staphylae were more enriched than hyphae. Letters show groupings based on pairwise tests (*p*_adj_ < 0.05). (*b*) An *in vivo* experiment showed that staphylae and hyphae were significantly ^13^C-enriched after 2 days compared to baseline natural abundance with no significant differences between fungal tissues. Z-scores relative to control/baseline, diamonds indicate means.

Fungal tissue was significantly enriched for ^13^C in both the ^13^C-cellulose and ^13^C-glucose treatments relative to the control (*F*_2,152_ = 18.487, *p* < 0.001, figure 1*a*). The cultivar responded similarly for both treatments with overall enrichment levels that did not differ statistically, and with staphylae being more enriched than hyphae (*F*_1,152_ = 24.168, *p* < 0.001; figure 1*a*; electronic supplementary material, figure S1).

### (b) *In vivo* cellulose assimilation by an intact fungus garden

The fungus garden assimilated ^13^C-cellulose from the substrate collected by foragers and provisioned by gardeners inside the nest, as both hyphae and staphylae sampled from the middle layer of the fungus garden had significantly elevated ^13^C-content (*F*_1,12_ = 14.405, *p* = 0.003) relative to baseline natural abundances (figure 1*b*). Enrichment in staphylae and hyphae did not differ significantly from each other (overall: *F*_1,12_ = 0.162, *p* = 0.694; Day 2: *t*_12_ = −0.569, *p* = 0.580).

### (c) Cultivar mediated cellulose metabolism

**Figure 2.**
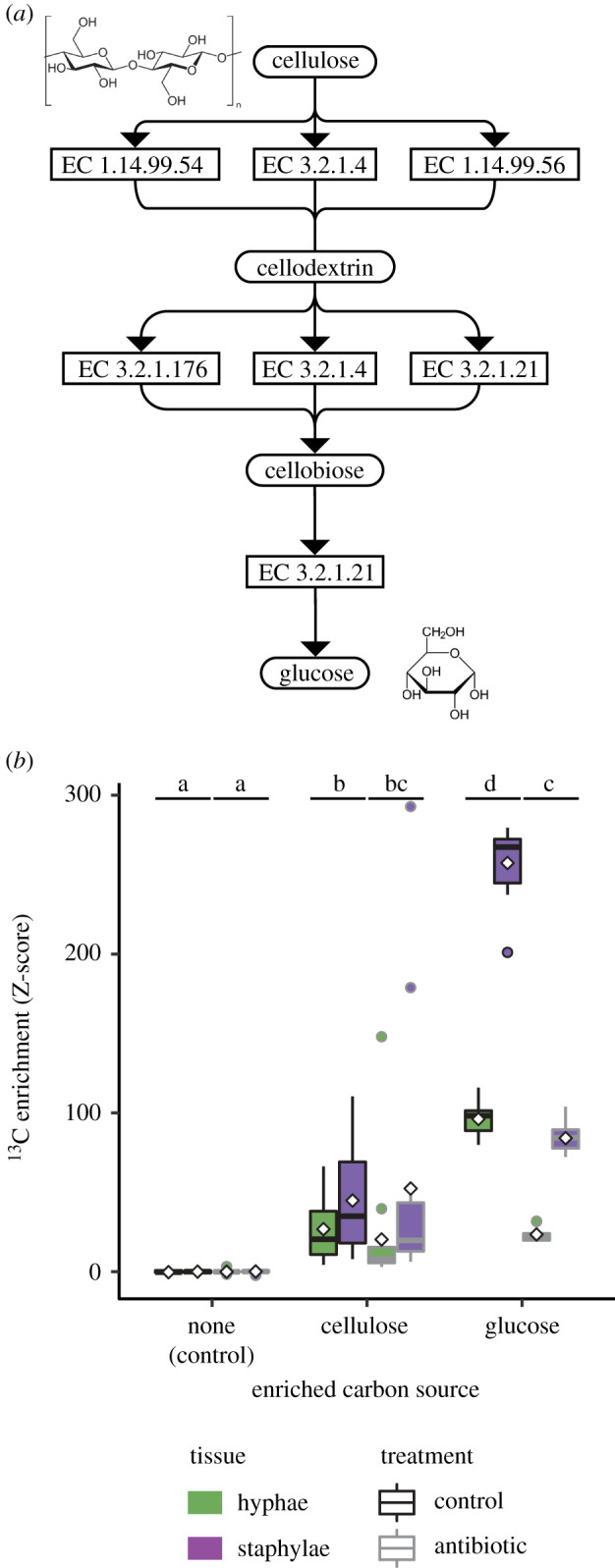
The fungal cultivar can directly metabolise and assimilate cellulose. (*a*) A complete cellulose-degradation pathway was identified from transcriptomic data. (*b*) The *in vitro* cultivar remained significantly ^13^C-enriched relative to the baseline natural abundance (control) when bacteria were specifically excluded using antibiotics. Z-scores relative to control, diamonds indicate means, letters show groupings based on pairwise tests (*p*_adj_ < 0.05).

High-resolution *in silico* analysis of transcriptomic data [33] confirmed that *L. gongylophorus* expresses all enzymes required for cellulose degradation and that these genes are expressed in a PDA medium lacking cellulose (figure 2*a*), potentially indicating constitutive expression of these biodegradative pathways. In total, we identified three cellulase genes (EC:3.2.1.4), four lytic cellulose monooxygenases (C1-hydroxylating) (EC:1.14.99.54), one lytic cellulose monooxygenase (C4-dehydrogenating) (EC:1.14.99.56), two cellulose 1,4-β-cellobiosidases (reducing end) (EC:3.2.1.176) and two β-glucosidases (EC:3.2.1.21) (figure 2*a*).

The antibiotic assay excluded the possibility that bacterial symbionts were necessary for cellulose metabolism, as the cultivar was significantly enriched for ^13^C in both the ^13^C-cellulose and ^13^C-glucose treatments, relative to samples from control PDA plates (*F*_1,171_ = 130.114, *p* < 0.001, figure 2*b*). Pairwise tests showed higher ^13^C enrichment in the glucose treatment relative to the cellulose treatment, even as both were still significantly enriched relative to the control (figure 2*b*). Despite evidence for ^13^C enrichment when bacteria were excluded, overall ^13^C enrichment was lower on plates with antibiotics relative to the respective control plates with only ^13^C-cellulose or ^13^C-glucose (*F*_1,171_ = 48.295, *p* < 0.001, figure 2*b*). However, a significant interaction between carbon source and antibiotic treatment (*F*_2,171_ = 47.314, *p* < 0.001), and subsequent pairwise tests, indicated the main effect was driven by reductions in ^13^C enrichment in the glucose treatment and with no significant effect of the antibiotic treatment on the cellulose medium (figure 2*b*). Staphylae were significantly enriched relative to hyphae (*F*_1,171_ = 58.084, *p* < 0.001, figure 2*b*).

## 4. Discussion

While *L. gongylophorus* is known to degrade cellulose [10,12–14,19] (electronic supplementary material, table S1), our isotopic enrichment experiments provide the first empirical confirmation of the prediction that it also metabolizes and assimilates cellulose-derived carbon into nutritional reward structures for ant farmers. The fungal cultivar further expresses its own complete enzymatic pathway for the degradation of cellulose to glucose and can metabolically transform cellulose following the targeted *in vitro* removal of bacteria (and ant farmers). The cultivar's metabolic conversion of cellulose to glucose and packaging in edible nutritional rewards may have contributed to the dietary niche expansion that has made leafcutter ants dominant herbivores across neotropical ecosystems.

Like free-living fungi [18], *L. gongylophorus* has been predicted to preferentially metabolize simple sugars over recalcitrant plant compounds like cellulose [9,10,12,13], with some further predicting that cellulase expression serves to degrade the plant cell wall rather than releasing usable carbon for the fungus [9,10,12,13]. However, the cultivar in this study metabolized cellulose despite having access to the simple sugar dextrose, at a concentration approximately 200 times higher than cellulose in the PDA medium. Transcriptomic analysis further identified expressed cellulase genes despite being collected from cultivars grown on cellulose-free PDA [33]. The ubiquity of cellulose in plant tissues may have favoured the evolution of a constitutive cellulose metabolism even when the individual fragments foraged contain this molecule at low concentrations, with cellulase production having been shown to increase in the presence of fresh plant material [13]. It will be interesting to perform differential-expression analyses testing whether cellulose gradients in provisioned substrates directly mediate cellulase gene expression levels and ultimately govern behavioural decisions in the colony about sending foraged leaf material directly to waste piles.

These results shed light on cellulose processing within *L. gongylophorus* fungus gardens. Fungal cellulase expression appears highest in the top and bottom layers of the garden [9,10,12], and the cultivar is assumed to only prioritize cellulose digestion once highly degraded plant material reaches the bottom layer [10,12,13]. Our *in vivo* results indicate that freshly foraged cellulose can be rapidly (within 2 days) converted into edible gongylidia in the middle layer, perhaps assisted by the constitutive expression of cellulase. Although we do not observe differential enrichment between staphylae and the surrounding mycelium in the *in vivo* experiment, our sampling point was based on rapidly assimilated glucose, with more complex substrates like cellulose potentially taking longer. Our results are based on a single attine cultivar, but we predict that this process of cellulose assimilation will hold across cultivars of other leafcutter colonies, species and genera, as their cultivars exhibit high degrees of relatedness [37]. Moreover, results of the present study add to an expanding catalogue of adaptions [6,27,33] enabling the domesticated fungal cultivar to extract nutrition from taxonomically and biochemically diverse plant fragments [20].

## Data Availability

All data generated in this study are available in the electronic supplementary material, table S2 and table S3 [38].
